# Regenerative strategies for surfactant deficiency in neonatal and pediatric lung disease: the role of mesenchymal stem cells and their extracellular vesicles

**DOI:** 10.3389/fcell.2026.1831705

**Published:** 2026-07-09

**Authors:** Gloria Pelizzo, Valeria Calcaterra, Maria Antonietta Avanzini, Erika Cordaro, Carlotta Ardenghi, Gianvincenzo Zuccotti

**Affiliations:** 1 Department of Biomedical and Clinical Science, University of Milan, Milan, Italy; 2 Pediatric Surgery Department, Buzzi Children’s Hospital, Milan, Italy; 3 Pediatrics and Adolescentology Unit, Department of Internal Medicine, University of Pavia, Pavia, Italy; 4 Pediatric Department, Buzzi Children’s Hospital, Milan, Italy; 5 Cell Factory and Center for Advanced Therapies, Fondazione IRCCS Policlinico San Matteo, Pavia, Italy

**Keywords:** children, extracellular vesicles, lung disease, mesenchymal stem cells, neonates, pediatrics, regenerative medicine, surfactant deficiency

## Abstract

**Introduction:**

Surfactant deficiency underlies several neonatal and pediatric lung diseases, including respiratory distress syndrome (RDS), bronchopulmonary dysplasia (BPD), and genetic surfactant disorders. Although advances in neonatal care and surfactant replacement therapy have improved survival in preterm infants, current treatments mainly provide supportive management and do not fully address mechanisms of lung injury and impaired lung development. Regenerative approaches based on mesenchymal stromal cells (MSCs) and their extracellular vesicles (EVs) have emerged as potential strategies to promote lung repair and restore alveolar homeostasis.

**Methods:**

This narrative review summarizes experimental and clinical evidence on MSC- and EV-based regenerative strategies for surfactant-related lung diseases. Relevant studies investigating MSCs or MSC-derived EVs in neonatal and pediatric lung injury were identified through searches in PubMed, Scopus and Web of Science and were qualitatively analyzed.

**Main evidence:**

Preclinical studies show that MSCs reduce inflammation, protect type II alveolar epithelial cells and promote alveolar development in models of immature lung injury. These effects are largely mediated by paracrine mechanisms, including the release of MSC-derived EVs, which transfer bioactive molecules to target cells and regulate inflammatory and regenerative pathways. Accordingly, EV-based therapies, as a cell-free derivative of MSCs, reproduce several beneficial effects of MSC transplantation in animal models. Early clinical studies in preterm infants at risk of BPD show encouraging safety profiles, although evidence of long-term efficacy remains limited.

**Conclusion:**

MSC- and EV-based regenerative approaches represent promising strategies for surfactant-related lung diseases. Further studies are needed to clarify mechanisms of action and evaluate their efficacy in well-designed clinical trials involving neonatal and pediatric populations.

## 1 Introduction

Pulmonary surfactant is essential for alveolar stability, gas exchange, and lung mechanics. Beyond its biophysical role, it contributes to host defense and regulation of inflammatory responses in the distal lung (Nkadi et al., 2009; Han and Mallampalli, 2015; Mason and Dobbs, 2016; Van Haaften et al., 2009).

Surfactant deficiency represents a major cause of respiratory morbidity and mortality in neonates and children. In preterm infants, insufficient surfactant synthesis due to lung immaturity leads to neonatal respiratory distress syndrome (RDS), characterized by impaired gas exchange, diffuse atelectasis and the need for respiratory support (Nkadi et al., 2009). Prolonged exposure to oxygen therapy and mechanical ventilation may further contribute to bronchopulmonary dysplasia (BPD), which is characterized by impaired alveolar and vascular development and, in contemporary neonatal care, by simplified alveolar structure and abnormal pulmonary vascular growth (Cicalò et al., 2023; Teng et al., 2025; Price et al., 2025; Cosgrove et al., 2025).

Surfactant dysfunction may also result from genetic defects in surfactant metabolism. Mutations in SFTPB, SFTPC, ABCA3**,** or NKX2-1 can cause severe neonatal respiratory failure or childhood interstitial lung disease (ChILD) (Nathan et al., 2023; Nevel et al., 2025). Together, these conditions emphasize the central role of alveolar type 2 (ATII) epithelial cells and surfactant homeostasis in maintaining normal lung function.

Although surfactant replacement therapy has improved neonatal outcomes, it remains largely supportive and does not fully address lung injury, impaired repair, or altered alveolar development. This has increased interest in regenerative approaches aimed at promoting lung repair and restoring alveolar homeostasis.

Among emerging regenerative strategies, mesenchymal stromal cells (MSCs) have attracted considerable attention because of their immunomodulatory and reparative properties. MSCs were first described by Friedenstein et al. (1968) as plastic-adherent stromal cells capable of forming fibroblast-like colonies in bone marrow cultures. They can be isolated from multiple tissues, including bone marrow, umbilical cord blood, placenta, adipose tissue and amniotic fluid, and are defined by plastic adherence, expression of CD105, CD73 and CD90, absence of hematopoietic markers, and the ability to differentiate into osteogenic, adipogenic and chondrogenic lineages (Dominici et al., 2006).

Although initially thought to act through direct differentiation, accumulating evidence indicates that MSC therapeutic effects are mainly mediated through paracrine mechanisms involving the release of cytokines, growth factors and anti-apoptotic mediators (Gnecchi et al., 2020; Trigo et al., 2025). In experimental models of neonatal lung injury, MSC administration attenuates inflammation, promotes alveolarization and improves pulmonary vascular development (Van Haaften et al., 2009). MSCs can also transfer functional mitochondria to injured cells via tunneling nanotubes or extracellular vesicles (EVs), thereby restoring cellular bioenergetics and enhancing survival (Tan et al., 2022; Chen et al., 2025).

Increasing attention has therefore focused on the MSC secretome and particularly on EVs, which are now considered key mediators of MSC paracrine activity (Trigo et al., 2025). EVs are membrane-bound nanoparticles released by most cell types that mediate intercellular communication through the transfer of proteins, lipids and nucleic acids (Jeppesen et al., 2024). MSC-derived EVs contain a complex molecular cargo, including mRNAs, microRNAs and bioactive proteins capable of modulating gene expression and cellular responses in recipient cells (Payandeh et al., 2024). Through these mechanisms, EVs regulate inflammatory pathways, support epithelial and endothelial regeneration and promote angiogenesis. Importantly, EV-based therapies may reproduce many beneficial effects of MSC transplantation while potentially overcoming limitations associated with cell-based therapies, including safety concerns and manufacturing challenges (Shimizu et al., 2026).

Given the key role of surfactant-producing ATII cells in neonatal lung development and injury, and their function as progenitor cells for alveolar epithelial repair, regenerative strategies aimed at preserving ATII function, restoring surfactant homeostasis, and promoting alveolar repair are promising therapeutic approaches for surfactant-related lung diseases.

In this narrative review, we summarize current knowledge on regenerative strategies for surfactant deficiency in neonatal and pediatric lung diseases, focusing on MSCs and their EVs. We discuss the biological rationale for their use in the developing lung, the mechanisms through which MSCs and EVs may support epithelial and vascular repair, and the available preclinical and clinical evidence supporting their potential translational application.

## 2 Methods

This review was conducted as a narrative literature review aimed at summarizing and qualitatively discussing the current evidence on regenerative strategies for surfactant deficiency in neonatal and pediatric lung diseases, with particular focus on MSCs and their EVs. A comprehensive literature search was performed using the PubMed, Scopus, and Web of Science databases. The search strategy combined keywords related to surfactant deficiency, neonatal lung disease, and regenerative therapies using Boolean operators (AND, OR). The main search terms included “surfactant deficiency,” “pulmonary surfactant,” “neonatal respiratory distress syndrome,” “RDS,” “bronchopulmonary dysplasia,” “pediatric lung disease,” “preterm infant,” “mesenchymal stem cells,” “extracellular vesicles,” “exosomes,” and “regenerative therapy.” Articles published in English up to 2025 were considered for inclusion, and the last literature search was performed in January 2026. In addition, the reference lists of relevant articles and review papers were manually screened to identify additional studies of interest.

The initial database search retrieved 462 records. After removal of duplicates and preliminary screening of titles and abstracts, 328 articles were excluded because they were not relevant to neonatal or pediatric lung diseases, did not address surfactant deficiency, or did not investigate regenerative approaches based on MSCs or EVs. The full texts of 134 articles were subsequently assessed for eligibility. Following full-text evaluation, 72 studies were excluded due to lack of relevance to the pediatric population, absence of experimental or clinical data on MSCs or EVs, or insufficient focus on lung injury or surfactant-related mechanisms. Ultimately, 62 studies were considered eligible and included in the qualitative synthesis of this review.

Eligible studies included both preclinical and clinical investigations relevant to MSCs or MSC-derived EVs in neonatal or pediatric lung injury. We considered studies addressing surfactant metabolism, alveolar epithelial injury, lung immaturity, and respiratory diseases associated with surfactant deficiency. Relevant review articles were also examined to identify key references and landmark studies.

Given the narrative nature of this review, no formal systematic review protocol or quantitative quality assessment was applied. Selected studies were qualitatively organized into thematic sections covering surfactant-related disease mechanisms, biological rationale, and preclinical and clinical evidence supporting MSC- and EV-based regenerative approaches.

## 3 Pulmonary surfactant and diseases related to surfactant deficiency

### 3.1 Physiology and functional role of pulmonary surfactant

Pulmonary surfactant is a specialized lipoprotein complex that lines the alveolar surface and plays a crucial role in maintaining lung stability and efficient gas exchange after birth. By reducing surface tension at the air–liquid interface of the alveoli, surfactant prevents end-expiratory alveolar collapse, improves lung compliance, and reduces the work of breathing (Han and Mallampalli, 2015; Mason and Dobbs, 2016).

Surfactant is synthesized and secreted by ATII, where it is assembled and stored in lamellar bodies before being released into the alveolar space (Nkadi et al., 2009). Once secreted, it spreads over the alveolar lining fluid to form a dynamic surface-active film that adapts to the cyclic changes in alveolar surface area during respiration (Han and Mallampalli, 2015).

The surfactant complex is composed predominantly of lipids (approximately 80%–90%), mainly phospholipids, together with surfactant-associated proteins and smaller amounts of neutral lipids such as cholesterol (Peers et al., 2023). Dipalmitoylphosphatidylcholine (DPPC) represents the principal surface-active phospholipid responsible for achieving extremely low surface tension during alveolar compression. Other phospholipids, including phosphatidylglycerol and unsaturated phosphatidylcholine species, contribute to membrane fluidity and facilitate rapid spreading of the surfactant film (Peers et al., 2023).

Four surfactant proteins (SP-A, SP-B, SP-C and SP-D) play key functional roles. The hydrophobic proteins SP-B and SP-C are essential for adsorption and stabilization of the surfactant film at the air–liquid interface, whereas SP-A and SP-D belong to the collectin family and participate in innate immune defense by promoting pathogen recognition and modulating inflammatory responses within the lung (Liekkinen et al., 2023).

Efficient surfactant function depends on tightly regulated processes of synthesis, secretion, recycling and degradation. In the immature lung, particularly in preterm infants, these mechanisms may be incompletely developed, rendering the alveolar environment vulnerable to surfactant dysfunction and respiratory failure.

### 3.2 Pathogenetic mechanisms of surfactant deficiency

Surfactant deficiency in neonatal and pediatric lung disease results from multiple mechanisms that may affect surfactant production, composition, function, or clearance. From a clinical perspective, these mechanisms should be prioritized according to their relative frequency and impact: developmental immaturity in preterm infants represents the leading cause, followed by genetic surfactant disorders, impaired surfactant clearance, and secondary surfactant inactivation due to inflammatory or injurious conditions. Qualitative alterations of surfactant composition and metabolism also play a major role (Nkadi et al., 2009; Han and Mallampalli, 2015; Mason and Dobbs, 2016).

In premature neonates, incomplete maturation of ATII cells leads to insufficient surfactant synthesis and storage, resulting in elevated alveolar surface tension and impaired lung compliance (Nkadi et al., 2009). These alterations contribute to respiratory distress shortly after birth and represent the primary mechanism underlying neonatal respiratory distress syndrome.

However, surfactant dysfunction may also arise from intrinsic defects of surfactant metabolism (Nevel et al., 2025; Peers et al., 2023). Monogenic disorders involving genes encoding surfactant proteins (SFTPB and SFTPC), lipid transporters such as ABCA3, or transcriptional regulators of lung development (e.g., NKX2-1) disrupt critical pathways including lamellar body formation, protein processing and phospholipid transport (Nevel et al., 2025). Severe mutations in SFTPB or ABCA3 typically cause life-threatening respiratory failure in the neonatal period due to profoundly abnormal surfactant composition. In contrast, dominant mutations in SFTPC often lead to misfolded protein accumulation and endoplasmic reticulum stress within ATII cells, resulting in epithelial injury and progressive interstitial lung disease (Nevel et al., 2025; Peers et al., 2023).

Defective surfactant clearance represents another important pathogenetic mechanism. In pulmonary alveolar proteinosis, impaired signaling through granulocyte–macrophage colony-stimulating factor (GM-CSF) leads to dysfunctional alveolar macrophages and accumulation of surfactant material within the alveolar space (Bendstrup et al., 2025).

Finally, surfactant function may be secondarily impaired by inflammatory or injurious conditions. Infection, hyperoxia, mechanical ventilation, and meconium aspiration can alter surfactant through oxidative damage, proteolytic degradation, phospholipase activity, or inhibition by plasma proteins that leak into the alveolar space during inflammation. In these situations, surfactant dysfunction reflects not only reduced production but also profound alterations of the alveolar microenvironment (Nkadi et al., 2009; Han and Mallampalli, 2015; Mason and Dobbs, 2016; Bendstrup et al., 2025).

Overall, surfactant deficiency should therefore be considered a multifactorial process involving developmental immaturity, epithelial injury, dysregulated immune responses, and disturbances in surfactant metabolism.

### 3.3 Main clinical conditions associated with surfactant dysfunction

#### 3.3.1 Neonatal respiratory distress syndrome (RDS)

Neonatal RDS represents the most common clinical manifestation of primary surfactant deficiency and remains a major cause of respiratory morbidity and mortality in preterm infants. The incidence of RDS is strongly inversely related to gestational age and birth weight, reflecting the developmental immaturity of ATII and the limited surfactant pool available at birth (Pattnaik et al., 2026).

Elevated alveolar surface tension leads to diffuse atelectasis, impaired gas exchange and reduced lung compliance, which in turn promote hypoxemia, respiratory acidosis and the formation of proteinaceous hyaline membranes within the alveoli. Clinically, affected infants typically present with tachypnea, grunting, nasal flaring and intercostal retractions shortly after birth (Holme and Chetcuti, 2012).

Advances in perinatal care, including antenatal corticosteroids, early CPAP and exogenous surfactant replacement therapy, have significantly improved survival rates in preterm infants with RDS (Kaluarachchi et al., 2026). Nevertheless, RDS remains an important contributor to neonatal morbidity and may represent the initial step in a continuum of lung injury that predisposes infants to chronic respiratory conditions such as bronchopulmonary dysplasia (Kaluarachchi et al., 2026).

Recent consensus statements and clinical guidelines emphasize the importance of early surfactant administration combined with lung-protective ventilation strategies in order to minimize ventilator-induced lung injury and improve long-term respiratory outcomes (Sweet et al., 2023).

#### 3.3.2 Genetic surfactant dysfunction disorders

Inherited disorders affecting surfactant metabolism represent a rare but increasingly recognized group of pediatric lung diseases. These conditions may present either as severe respiratory failure in term or late-preterm neonates or as chronic diffuse lung disease during infancy or childhood (Nevel et al., 2025).

Pathogenic variants in genes encoding surfactant-related proteins and transporters, most notably SFTPB, SFTPC, ABCA3 and NKX2-1, disrupt critical pathways involved in surfactant synthesis, processing and intracellular trafficking within ATII (Nevel et al., 2025).

Loss-of-function mutations in SFTPB typically result in severe congenital surfactant deficiency with rapidly progressive respiratory failure that is often fatal without lung transplantation. Mutations in ABCA3**,** a phospholipid transporter required for lamellar body formation, represent one of the most common genetic causes of congenital surfactant dysfunction and may lead to severe neonatal respiratory distress or pediatric interstitial lung disease (Nevel et al., 2025; Peers et al., 2023).

In contrast, SFTPC mutations usually act through toxic gain-of-function mechanisms involving misfolded protein accumulation and endoplasmic reticulum stress in ATII cells. These alterations can cause epithelial injury and chronic interstitial lung disease with highly variable clinical presentation. Recent cohort analyses have also suggested genotype-dependent differences in clinical severity and prognosis among patients with surfactant dysfunction disorders (Papiris et al., 2022).

More broadly, genetic surfactant disorders are now recognized as part of the spectrum of childhood interstitial lung diseases (chILD) and represent an important diagnostic consideration in infants presenting with unexplained respiratory failure or persistent diffuse lung disease.

#### 3.3.3 Bronchopulmonary dysplasia

BPD is the most common chronic lung disease of prematurity and arises from the interaction between developmental lung immaturity and postnatal injury (Cicalò et al., 2023; Cosgrove et al., 2025). Although BPD is not primarily caused by surfactant deficiency, surfactant dysfunction contributes to its pathogenesis, particularly during the early stages of disease development.

Extremely preterm infants are frequently exposed to multiple injurious factors, including hyperoxia, mechanical ventilation, infection and systemic inflammation, during critical phases of lung development (Obst et al., 2022). These exposures disrupt normal alveolar and vascular growth and interfere with epithelial cell function.

In the current era of neonatal care, the so-called “new BPD” is characterized predominantly by arrested alveolarization, reduced pulmonary microvascular development and simplified lung architecture rather than the extensive fibrosis observed in earlier forms of the disease (Cicalò et al., 2023; Cosgrove et al., 2025). These structural abnormalities contribute to long-term respiratory impairment and increased susceptibility to chronic lung disease later in life.

Surfactant dysfunction in BPD is therefore best understood as part of a broader disturbance of the epithelial–vascular unit in the immature lung, involving inflammation, oxidative stress and impaired repair mechanisms.

#### 3.3.4 Childhood interstitial lung disease (ChILD)

ChILD represent a heterogeneous group of rare respiratory disorders affecting infants and children (Tonazzo et al., 2026). More than 200 conditions are included within the ChILD spectrum, many of which involve abnormalities in surfactant metabolism, epithelial cell injury or impaired lung development (Tonazzo et al., 2026).

These disorders are characterized by diffuse inflammation and remodeling of the lung parenchyma that may progressively evolve toward respiratory insufficiency and pulmonary fibrosis. Clinical manifestations often include persistent tachypnea, hypoxemia, growth impairment and reduced exercise tolerance.

Genetic mutations affecting surfactant-related genes represent an important subgroup within ChILD and are frequently associated with more severe disease courses. Recent genomic studies indicate that numerous genes involved in surfactant metabolism, epithelial homeostasis and immune regulation contribute to the pathogenesis of pediatric interstitial lung disease (Tonazzo et al., 2026).

Therapeutic options for ChILD remain limited and are largely supportive. Corticosteroids, hydroxychloroquine and immunomodulatory agents are sometimes used, but evidence for their efficacy is limited and controlled clinical trials in pediatric populations are scarce (Tonazzo et al., 2026). These limitations highlight the need for novel therapeutic strategies capable of promoting epithelial repair and restoring alveolar homeostasis.

### 3.4 Limitations of conventional therapies

Exogenous surfactant replacement therapy has dramatically improved outcomes in neonatal respiratory distress syndrome by restoring alveolar surface activity and stabilizing lung mechanics. Early administration in high-risk preterm infants improves oxygenation, lung compliance and survival (Kaluarachchi et al., 2026). Despite these benefits, conventional surfactant therapy has several limitations (Wilson et al., 2015).

First, it mainly corrects the biophysical consequences of surfactant deficiency without addressing the underlying cellular and developmental abnormalities of the immature lung. In particular, surfactant administration does not restore ATII cell function nor prevent the inflammatory and structural remodeling that contributes to chronic lung disease.

Second, surfactant replacement therapy is ineffective in disorders caused by intrinsic defects in surfactant metabolism, such as SFTPB or ABCA3 deficiency (Peers et al., 2023). In these conditions the fundamental defect lies in surfactant synthesis, processing or intracellular trafficking rather than in the absence of surfactant within the alveolar space.

Third, in inflammatory lung injury both endogenous and administered surfactant may become functionally inactivated (Hentschel et al., 2020). Oxidative stress, proteolytic enzymes and leakage of plasma proteins into the alveoli can disrupt the surfactant film and reduce its surface-active properties.

Finally, technical challenges remain. Surfactant is usually delivered through endotracheal instillation, an invasive procedure that may be associated with transient complications such as hypoxemia or airway obstruction (Mani and Rawat, 2024). Less invasive delivery strategies are currently under investigation but have not yet fully replaced traditional administration methods.

Taken together, these limitations indicate that surfactant replacement therapy, although highly effective in acute RDS, does not fully restore alveolar homeostasis or promote regeneration of the injured developing lung. This has stimulated increasing interest in regenerative strategies aimed at protecting ATII cells, modulating inflammation and promoting lung repair (Hentschel et al., 2020).

In this context, MSCs and their EVs have emerged as promising therapeutic candidates capable of modulating inflammatory responses and supporting epithelial regeneration in neonatal lung injury.

## 4 Rationale for the use of mesenchymal stem cells and extracellular vesicles

### 4.1 Biological rationale in the immature lung

The immature lung represents a developmentally dynamic organ in which structural maturation, epithelial differentiation, and vascular growth occur within a narrow and highly vulnerable temporal window (Kang and Thébaud, 2018). Perinatal complications may permanently compromise coordinated alveolar and vascular development, inducing long-term injuries to the lung (Van Haaften et al., 2009). In this context, increasing attention has been directed toward the regenerative properties and therapeutic potential of MSCs and MSC-derived secretome-based approaches (Van Haaften et al., 2009).

#### 4.1.1 MSC-based evidence

Different studies have demonstrated that injected bone marrow-derived MSCs differentiate into parenchymal cells in various non-hematopoietic tissues, including the lung (Phinney and Prockop, 2007; Chang et al., 2009; Ahn et al., 2013). In preclinical neonatal hyperoxia models, systematic analyses have reported that MSCs and MSC-conditioned media improve alveolarization and angiogenesis while reducing pulmonary artery remodeling (Augustine et al., 2017). (Van Haaften et al., 2009) further supported this rationale in a chronic hyperoxia-induced model of BPD in newborn rats. Hyperoxia exposure caused air-space enlargement, arrested alveolarization, and reduced circulating and lung-resident colony-forming unit–fibroblasts, whereas bone marrow CFU-F were preserved. Bone marrow-derived MSCs also migrated preferentially toward injured lung tissue and, in co-culture experiments, expressed SP-C and showed lamellar body-like ultrastructural features (Van Haaften et al., 2009). *In vitro*, BMSC-derived conditioned medium prevented oxygen-induced DNA damage and apoptosis in fetal ATII, accelerated ATII wound healing, and enhanced endothelial network formation under hyperoxic conditions (Van Haaften et al., 2009) although long-term benefit may be greater after MSC treatment than after MSC-conditioned medium alone (Sutsko et al., 2013). Similarly, (Gülaşı et al., 2016) reported that tracheally administered MSCs were detectable in hyperoxia-exposed neonatal rat lungs and co-expressed SP-C, suggesting interaction with, or partial adoption of, ATII-associated features *in vivo*.

#### 4.1.2 EV-based evidence

Recent studies have emphasized the role of EVs in neonatal lung development and injury. EVs are membrane-bound particles ranging from 50 nm to 1 μm that originate from the endosomal or plasma membrane and mediate intercellular communication through molecular cargo transfer (van Niel et al., 2018). Ransom et al. (2023) showed that EVs are detectable in tracheal aspirates of premature infants as early as 22 weeks’ gestation, supporting the concept that vesicle-mediated signaling is active during lung development. EV-enriched tetraspanins, including Cd9, Cd63, and Cd81, are differentially expressed across epithelial subpopulations, with ATII preferentially expressing Cd63, whereas Cd9 and Cd81 are more highly expressed in AT1 (Ransom et al., 2023). These findings provide a developmental rationale for investigating EVs as mediators and potential therapeutic tools in immature lung injury (Ransom et al., 2023).

The therapeutic effects of MSC-EVs have been investigated in neonatal rodent models using intravenous, intraperitoneal, and intratracheal administration (Willis et al., 2017; Ahn et al., 2018; Chaubey et al., 2018; Willis et al., 2018a). Because premature newborns often receive intratracheal therapies, this route may be particularly relevant for targeting the injured lung. Porzionato et al. (2019) showed that intratracheally administered MSC-EVs reduced hyperoxia-induced lung damage and improved alveolarization and pulmonary vascular remodeling in a neonatal rat model of BPD. Notably, EVs appeared more effective on alveolarization than their cells of origin, although the authors emphasized the need for further studies on EVs quantification, characterization, biodistribution, and toxicity (Porzionato et al., 2019). These findings were subsequently reinforced in a neonatal hyperoxia model followed by normoxic recovery (Porzionato et al., 2021).

#### 4.1.3 ATII and surfactant linkage

The beneficial effects of MSC-based therapies appear unlikely to depend solely on direct engraftment. Van Haaften et al. (2009) proposed that the magnitude of structural and functional improvement after MSC administration exceeds what would be expected from engraftment alone, supporting paracrine-mediated mechanisms as a major therapeutic driver. This is particularly relevant for ATII, which represent both progenitor cells for the distal alveolar epithelium and the main source of pulmonary surfactant.

Within this framework, vesicle-based interventions may intersect with surfactant homeostasis. Zhou et al. (2022) reported that microvesicle administration partially restored SP-C protein levels, the major surfactant protein synthesized by ATII cells, whereas no restoration was observed for the other surfactant proteins analyzed, namely SP-A1, SP-B, and SP-D. You et al. (2020) further showed that human umbilical cord MSC-derived small EVs increased SP-C and CD31 staining in hyperoxia-exposed neonatal rats, supporting effects on both epithelial and endothelial compartments. In the same study, EVs treatment reduced TUNEL-positive lung cells, increased Ki-67-positive cells, downregulated PTEN and cleaved caspase-3, and upregulated phosphorylated Akt and VEGF-A, linking tissue protection to cell-survival and angiogenic pathways (You et al., 2020).

Taken together, these findings support a biological rationale in which neonatal lung injury involves disrupted distal lung development, impaired endogenous repair, altered vesicle-mediated epithelial communication, and vulnerability of the epithelial–vascular unit. In this setting, ATII dysfunction is particularly relevant because it may simultaneously impair surfactant synthesis and limit epithelial regeneration, thereby linking surfactant deficiency with defective alveolar repair. Within this framework, MSC- and secretome-based regenerative strategies emerge as mechanistically coherent approaches in surfactant-deficiency–related neonatal lung disease contexts.

### 4.2 Mechanisms of action of MSCs and EVs

At a mechanistic level, MSC- and EV-based therapies can be conceptualized as a coordinated sequence of events in which immunomodulation represents an early step, followed by protection of the alveolar epithelium and preservation of the epithelial–vascular unit, ultimately contributing to improved lung repair and alveolar development.

#### 4.2.1 Immunomodulation

A systematic review and meta-analysis of preclinical studies (Augustine et al., 2017) on neonatal hyperoxia models show that MSCs administration has significant therapeutic benefits. It ameliorates pulmonary hypertension and attenuates lung inflammation, fibrosis, and apoptosis, while improving angiogenesis. The same systematic review reports that MSC-conditioned media exert comparable effects on alveolarization and angiogenesis, supporting the concept that secreted factors mediate inflammatory modulation. Another meta-analysis focusing specifically on MSC-conditioned media across lung disease models also reported significant reductions in inflammatory cells and inflammatory cytokines, with effect sizes comparable to those observed with MSCs therapy (Emukah et al., 2019). Collectively, these findings suggest that the therapeutic effects of MSC-based approaches are largely mediated by paracrine mechanisms that regulate inflammatory responses and support lung repair. Zhou et al. showed, in a rat model of antenatal lipopolysaccharide (LPS)-induced bronchopulmonary dysplasia, that human umbilical cord MSC-derived microvesicles reduced macrophage infiltration and attenuated lung inflammation, with modulation of cytokine levels, including IL-6 and IL-10 (Zhou et al., 2022). Data also showed that MV treatment suppressed the expression levels of p-p38, p-JNK, and p-ERK. As ERKs, JNKs, and p38 MAPK are signaling mediators of MAPK and MAPK plays a role in the response to inflammation, the authors suggest that MVs can potentially alleviate LPS-induced lung injuries by a mechanism associated with the suppressed MAPK pathway. The study conducted by Porzionato et al. (2021) showed that intratracheal MSC-derived EV administration restores CD163-positive macrophage populations, consistent with preservation of an M2-like reparative phenotype in the injured lung, even if no significant differences in interstitial, alveolar and perivascular F4/8-positive and CD86-positive macrophages were detected. However, this selective macrophage polarization occurs in parallel with structural improvement, and supports the immunomodulation potential. Finally, a review focused on exosome-based therapeutics in neonatal lung injury also identifies regulation of inflammatory pathways as a central mechanistic axis of EV-mediated benefit (Willis et al., 2018b). Together, these data indicate that immunomodulation represents a core mechanistic pillar of MSC- and EV-based regenerative strategies in neonatal lung injury models. By limiting inflammatory injury in the distal lung, MSC- and EV-mediated immunomodulation may indirectly preserve ATII viability and support surfactant homeostasis.

#### 4.2.2 Protection of the alveolar epithelium and distal lung architecture

As reported above, in the study conducted by Van Haaften et al. (2009) intratracheal MSC administration in neonatal hyperoxia models was shown to prevent arrested alveolar growth when compared to the untreated hyperoxia controls group, as evidenced by improved alveolar morphology. Furthermore, MSC treatment preserves pulmonary microvascular density and attenuates pulmonary hypertension, supporting protection of the integrated alveolar–vascular unit. The authors also report that MSC-conditioned medium was shown to prevent oxygen-induced apoptosis and DNA damage in fetal ATII and accelerates ATII wound closure *in vitro* when compared to ATII cultured in Dulbecco’s modified Eagle’s medium (DMEM), demonstrating direct epithelial-protective effects mediated by secreted factors. Finally, it enhances endothelial cord formation under hyperoxic conditions, indicating preservation of endothelial function relevant to distal lung development. (Porzionato et al., 2021) confirm these data in their study, reporting that intratracheal administration of GMP-produced MSC-EVs preserves total alveolar number, alveolar surface area, and proliferation index while reducing mean alveolar volume, mean linear intercept, and fibrosis percentage. Of note, these structural improvements occur despite low levels of cellular engraftment, reinforcing the primacy of secreted or vesicle-mediated mechanisms. The authors noted that MSC-EVs reduced the medial thickness of small pulmonary vessels, further supporting restoration of the alveolar–vascular interface. Taken together, these studies indicate that MSCs and MSC-derived EVs may protect distal lung architecture through coordinated effects on epithelial survival, vascular preservation, and extracellular matrix remodeling. Following the intraperitoneal injection of BMSC into experimental BPD mice on post-natal day 7, structural and functional benefits were noted by (Zhang et al., 2012). Particularly, a reduction in the pulmonary expression of a number of genes (such as TGFb1, collagen 1a and TIMP-1) involved in extracellular matrix remodeling and fibrosis were reported, with an improvement in pulmonary architecture and the attenuation of pulmonary fibrosis around the airways and alveoli, thus increasing the survival rate. Finally, (Chen et al., 2017) investigated the effects of a surfactant on the *in vitro* viability and *in vivo* function of human MSCs. The intratracheal administration of MSCs, surfactant, or surfactant + MSCs on postnatal day 5 led to alveolarization improvement in hyperoxia-exposed neonatal rats. When evaluating surfactant and MSC administration results, both MSCs alone and MSCs plus surfactant significantly reduce hyperoxia-induced increases in mean linear intercept and apoptotic cell burden. These findings support epithelial and architectural protection as an important outcome across neonatal MSC studies (Augustine et al., 2017). Protection of the alveolar epithelium is particularly relevant in surfactant-related lung disease, because preservation of ATII integrity is essential for both surfactant production and epithelial repair.

#### 4.2.3 Support of ATII

*In vitro* experiments have shown that BMSCs can develop immunophenotypic and ultrastructural characteristics of ATII, including SP-C mRNA expression and lamellar bodies (Van Haaften et al., 2009). In the same study, engrafted MSCs have been observed to co-express the ATII-specific marker SP-C *in vivo*, linking MSC localization with ATII-associated phenotypes. Similarly, GFP-positive MSCs detected in lung tissue following MSC administration in hyperoxia-exposed neonatal rats have been reported to assume the properties of ATII and start synthesizing surfactants, thus suggesting an interaction with the surfactant-producing compartment (Gülaşı et al., 2016). However, the authors underlined that these findings may also be due to co-localization of MSC and SP-C. However, the co-localization was observed in injured lung tissue, suggesting that MSC can differentiate into lung parenchymal cells but, as this was observed only in a few cells, further investigations are needed. In an antenatal inflammation model of experimental BPD, hUCMSCs derived microvesicles promote ATII cell proliferation and enhance alveolar development (Zhou et al., 2022). Moreover, a modulation in SP-C levels was reported, suggesting that EVs can directly support the surfactant-producing epithelial population. Analysis on EV characterization in preterm lungs further demonstrates that EV-associated markers such as CD63 are expressed also in ATII during canalicular and saccular gestational stages (Ransom et al., 2023), reinforcing the concept that vesicle-mediated signaling is intrinsically linked to surfactant-producing epithelial cells. Taken together, these data demonstrate that MSC- and EV-based strategies exert measurable effects on ATII-associated markers. Overall, these findings support the concept that MSC- and EV-based therapies may contribute to epithelial repair not only by reducing injury, but also by interacting with the surfactant-producing ATII compartment.

#### 4.2.4 Extracellular vesicles as a cell-free therapeutic alternative

EVs have emerged as an important component of the MSC secretome and are increasingly investigated as a cell-free therapeutic alternative to MSC administration in neonatal lung injury. Compared with whole-cell approaches, MSC-derived EVs may offer potential advantages related to safety, storage, dosing, and standardization, while preserving key paracrine activities relevant to lung repair (Augustine et al., 2017; Emukah et al., 2019; Willis et al., 2018b). In relation to other emerging regenerative strategies, such as organoid-based methods or biomaterial scaffolds, MSC- and EV-based therapies may provide more readily available and less complex approaches with reduced immunogenicity (Hoang et al., 2025; Torkashvand et al., 2024; Khayatan et al., 2025), although their long-term biodistribution and persistence in the developing lung remain incompletely defined.

In a mouse model of hyperoxia-induced lung injury, (You et al., 2020) reported that intratracheally administered MSC-derived small EVs were detectable in lung tissue for at least 72 h, indicating effective pulmonary delivery and local retention within the injured distal lung compartment. In the same study, EV treatment exerted protective effects on ATII and pulmonary vascular endothelial cells (PVECs), improving alveolar structure and attenuating pulmonary hypertension. The authors also highlighted the need for further studies to clarify the complex mechanisms underlying MSC-EVs–mediated repair. These observations suggest that EVs may recapitulate several beneficial effects of parental MSCs while offering a more controllable cell-free therapeutic platform.

Additional evidence comes from studies using neonatal hyperoxia models. Porzionato et al. showed that intratracheal administration of GMP-produced MSC-derived EVs preserved alveolar number, alveolar surface area and cellular proliferation while reducing mean linear intercept, fibrosis percentage and pulmonary vascular remodeling (Porzionato et al., 2021). Bisaccia et al. (2024) observed protection from oxidative stress and a reduction in fibrosis-associated outcomes following intratracheal administration of human clinical-grade MSC-derived EVs in hyperoxia-exposed rat pups. Lung morphological improvement was associated with decreased markers of oxidative stress, including 8-oxo-dG, as well as reduced collagen deposition, suggesting that MSC-EVs may interfere with pathological processes linking oxidative stress and fibrosis. The same study also reported increased epithelial proliferation and higher SP-C expression compared with untreated hyperoxia controls, supporting a beneficial effect on surfactant-producing epithelial compartments. These findings collectively indicate that EV-based approaches may promote epithelial regeneration and support surfactant-related pathways in the immature lung. In addition, (Ransom et al., 2023) reported that EV-enriched tetraspanins are dynamically expressed in epithelial subpopulations during the canalicular and saccular stages of lung development, including patterns associated with ATII markers. This observation further strengthens the biological rationale for EV-based therapeutic strategies in surfactant-deficiency–related lung disorders.

Overall, MSC- and EV-based therapies share common regenerative mechanisms but differ in several practical and translational aspects. MSCs provide a dynamic and responsive source of paracrine factors but may be associated with challenges related to cell engraftment, viability, and potential immunogenicity. In contrast, EVs represent a cell-free alternative with advantages in terms of safety profile, storage, standardization, and scalability, and may allow more controlled delivery of bioactive cargo. However, uncertainties remain regarding EV biodistribution, dosing, and long-term effects. Together, these approaches may be viewed as complementary strategies within the broader framework of regenerative therapies for neonatal lung disease.

## 5 Evidence from preclinical and clinical studies and current limitations

### 5.1 Preclinical studies with MSCs in models of lung injury and surfactant deficiency

Preclinical studies have provided important evidence supporting the regenerative potential of MSCs in neonatal lung injury, particularly in relation to impaired alveolar development, epithelial repair, and surfactant-related lung disease.

Studies in neonatal hyperoxia models show that MSC administration can improve alveolar development, reduce epithelial injury, and attenuate inflammation, fibrosis, apoptosis, and pulmonary vascular remodeling (Van Haaften et al., 2009; Gülaşı et al., 2016; Aslam et al., 2009; Tropea et al., 2012). These effects appear to be mediated mainly by paracrine mechanisms rather than stable engraftment or direct differentiation (Van Haaften et al., 2009; Aslam et al., 2009). MSC-conditioned medium has shown comparable protective effects in several models, supporting the role of secreted factors in lung repair (Van Haaften et al., 2009; Aslam et al., 2009).

Quantitative synthesis of the preclinical literature has further strengthened these observations. A systematic review and meta-analysis conducted by Augustine et al. (Augustine et al., 2017) showed that MSC administration significantly improved alveolarization, with an SMD of −1.33 (95% CI −1.72 to −0.94; I^2^ = 69%). MSCs also improved pulmonary hypertension (SMD −1.57, 95% CI −2.21 to −0.92), reduced lung fibrosis (SMD −2.55, 95% CI −3.95 to −1.14), enhanced angiogenesis (SMD −1.55, 95% CI −1.95 to −1.16), and reduced apoptosis (SMD −1.01, 95% CI −1.79 to −0.22). Despite these encouraging findings, several limitations remain. Most evidence derives from hyperoxia-induced rodent models, which only partially reproduce the complex pathophysiology of neonatal lung disease in extremely preterm infants. In addition, heterogeneity in MSC source, dose, timing of administration, and delivery route limits direct comparison across studies and highlights the need for standardized preclinical protocols. These preclinical findings are relevant to surfactant-deficiency–related disease because improved alveolarization and reduced epithelial injury may help preserve the ATII compartment, which is central to surfactant synthesis and alveolar repair.

### 5.2 Preclinical studies with extracellular vesicles

Preclinical studies have increasingly investigated MSC-derived EVs as a cell-free therapeutic approach in neonatal lung injury, supporting their potential role in immature lung repair and surfactant-related disease.

One of the first studies directly comparing MSCs and MSC-derived EVs in neonatal lung injury was conducted by (Porzionato et al., 2019) using a hyperoxia-induced rat model of BPD. In this study, intratracheal administration of MSC-derived EVs significantly reduced lung injury and improved alveolarization and pulmonary vascular development. Interestingly, EV treatment produced structural improvements comparable to, and in some parameters greater than, those observed following administration of the parental MSCs.

These results were extended in a subsequent study by (Porzionato et al., 2021) using a chronic hyperoxia model followed by normoxic recovery. Repeated intratracheal administration of GMP-produced MSC-derived EVs preserved alveolar number and surface area while reducing pulmonary fibrosis and vascular remodeling. The authors also observed preservation of CD163-positive macrophages, suggesting maintenance of a reparative macrophage phenotype in the injured lung.

Recent studies have further explored the therapeutic potential of EVs produced under clinical-grade manufacturing conditions. In a neonatal rat model of hyperoxia-induced lung injury, (Bisaccia et al., 2024) showed that intratracheal administration of human umbilical cord MSC-derived EVs reduced oxidative stress and fibrosis-related changes while supporting epithelial regeneration and surfactant-associated protein expression.

An important step toward translational relevance has been the use of large-animal models that better reproduce the physiological and clinical characteristics of extremely preterm infants. In a recent study, (Albertine et al., 2024) evaluated MSC-derived small EVs in mechanically ventilated preterm lambs and reported improved gas exchange, alveolar development, and pulmonary capillary density. These findings support the translational potential of EV-based therapies for immature lung injury.

Overall, preclinical studies indicate that EVs can reproduce many of the beneficial effects observed with MSC therapy, including improved alveolar development and attenuation of lung injury in immature lungs. In this context, EV-mediated effects on epithelial regeneration, inflammation, and vascular development should be interpreted not only as general lung-protective mechanisms, but also as processes potentially supporting ATII function and surfactant homeostasis. Across studies, these beneficial effects appear generally consistent, although variability in outcomes may reflect differences in animal species, injury models, timing of intervention, cell or EV sources, and delivery routes. However, several translational challenges remain. Experimental studies differ considerably in EV isolation techniques, characterization methods, dosing strategies and routes of administration. Moreover, key aspects such as EV biodistribution, pharmacokinetics and long-term safety remain incompletely understood. The main preclinical studies investigating MSC- and EV-based approaches in models of neonatal lung injury are summarized in Table 1.

**TABLE 1:** Preclinical MSC and EV studies in models of neonatal lung injury.

Reference	Model	Source	Key outcomes	Mechanism
Fang et al. (2010)	ATII + pro-inflammatory cytokines	hBM-MSC	Restoration of epithelial barrier permeability	Angiopoietin-1 secretion
Schwede et al. (2018)	ATII + pro-inflammatory cytokines	hBM-MSC	↓ inflammatory genes; ↑ surfactant protein B expression	↑ Periostin, Ang-1, EGFR, HGF signaling
Ai et al. (2022)	ATII + hyperoxia	hUC-MSC-EVs	↓ transdifferentiation from ATII to ATI	↓ WNT5a expression
Aslam et al. (2009)	Neonatal mice exposed to hyperoxia	mBM-MSC + CM	↓ inflammation; improved vascular structure	Immunomodulatory paracrine signaling
Tropea et al. (2012)	Newborn mice exposed to hyperoxia	mBM-MSC + CM	Activation of bronchioalveolar stem cells (BASCs)	Stem cell niche activation
Zhang et al. (2012)	Neonatal rats exposed to hyperoxia	rBM-MSC	↓ inflammation; ↓ cytokines; ↓ apoptosis	↑ AQP5, SP-C, BCL-2; ↓ BAX
Hansmann et al. (2012)	Mouse pups exposed to hyperoxia	mBM-MSC + CM	↓ fibrosis; ↓ pulmonary arterial muscularization	Anti-fibrotic paracrine signaling
Gülaşı et al. (2016)	Rat pups exposed to hyperoxia	rBM-MSC	↑ alveoli; ↓ α-SMA expression	Reduced myofibroblast activation
Sutsko et al. (2013)	Newborn rats exposed to hyperoxia	mBM-MSC + CM	↓ inflammatory cytokines; ↑ angiogenic factors; ↑ alveolarization	Paracrine angiogenic signaling
Benny et al. (2022)	Newborn rats exposed to hyperoxia	hUC-MSC-EVs/hBM-MSC-EVs	↑ alveolarization; protection from pulmonary hypertension	↑ pro-angiogenic genes
Porzionato et al. (2021)	Rat model exposed to hyperoxia	hUC-MSC-EVs	↓ fibrosis; ↑ alveolarization; improved pulmonary vascularization	↓ ROS; restoration of ATI/ATII ratio
Bisaccia et al. (2024)	Rat model exposed to hyperoxia	hUC-MSC-EVs	↓ oxidative stress and fibrosis; ↑ alveolarization	Anti-oxidative EV cargo
Willis et al. (2018a)	Neonatal mice exposed to hyperoxia	MSC-derived small EVs	Restoration of lung architecture; ↑ exercise capacity	Immunomodulation and epithelial repair
Van Haaften et al. (2009)	Neonatal rat lung injury model	BM-MSC	Prevention of arrested alveolar growth	Paracrine regenerative signaling
Moreira et al. (2020)	Experimental BPD model	UC-MSC	Restoration of alveolarization and vascularization	Lung regenerative signaling
Albertine et al. (2024)	Mechanically ventilated preterm lambs	BM-MSC-EVs	↑ alveolar count; ↑ capillary density; improved gas exchange	Epithelial and vascular repair
Möbius et al. (2023)	Extremely premature non-human primates	MSC	Safe administration; limited structural improvement	Development-stage dependent response

α-SMA: alpha-smooth muscle actin; ATI: type I alveolar epithelial cells; ATII: type II, alveolar epithelial cells; BASCs: bronchioalveolar stem cells; BPD: bronchopulmonary dysplasia; CM: conditioned medium; EVs: extracellular vesicles; hBM-MSC: human bone marrow–derived mesenchymal stromal cells; hUC-MSC-EVs: extracellular vesicles derived from human umbilical cord mesenchymal stromal cells; mBM-MSC: murine bone marrow–derived mesenchymal stromal cells; MSC: mesenchymal stromal cells; rBM-MSC: rat bone marrow–derived mesenchymal stromal cells; ROS: reactive oxygen species.

### 5.3 Clinical studies and evidence on MSCs and extracellular vesicles

Despite promising experimental results, preclinical evidence remains limited by the predominant use of rodent hyperoxia models, heterogeneity in MSC or EV sources, dosing, timing and delivery routes, and incomplete data on biodistribution, pharmacokinetics and long-term safety. These limitations should be considered when translating experimental findings into clinical practice.

The first clinical trial evaluating MSC therapy in preterm infants at high risk of BPD was conducted by Chang et al. (2014) **.** In this phase I dose-escalation study, nine extremely premature infants received intratracheal administration of human umbilical cord blood-derived MSCs. The treatment was well tolerated and no serious adverse events related to MSC therapy were observed. Importantly, analysis of tracheal aspirates demonstrated a reduction in inflammatory cytokines following MSC administration, suggesting a potential anti-inflammatory effect in the injured neonatal lung.

A subsequent follow-up study by (Ahn et al., 2017) evaluated the long-term safety of MSC therapy in the same patient cohort. The authors reported that MSC administration remained safe up to 2 years of age, with no evidence of tumor formation, immune reactions or other treatment-related complications.

Further clinical evaluation was performed in a randomized phase II clinical trial conducted by (Ahn et al., 2021), which investigated the efficacy of intratracheal administration of umbilical cord-derived MSCs for the prevention of severe BPD. Although the study population was relatively small, the results suggested a potential reduction in the incidence of severe BPD, particularly among the most immature infants, supporting the rationale for larger multicenter trials.

Clinical experience with MSC therapy has also been reported in pediatric patients with rare genetic lung disorders affecting surfactant metabolism. (Pelizzo et al., 2023) described the administration of repeated intravenous infusions of allogeneic bone marrow-derived MSCs in a child with progressive respiratory failure caused by a SP-C gene mutation. The treatment was well tolerated and associated with progressive improvement in respiratory function and reduced ventilatory support requirements. Although limited to a single case report, this observation suggests that MSC therapy may represent a potential therapeutic approach for pediatric interstitial lung diseases associated with surfactant dysfunction.

It is worth noting that MSC therapy has also been explored in several other clinical contexts not directly related to surfactant deficiency. In particular, MSCs have been investigated in immune-mediated conditions such as steroid-resistant graft-versus-host disease following hematopoietic stem cell transplantation, where MSC administration has demonstrated significant immunomodulatory effects and improved clinical outcomes (Le Blanc et al., 2004; Ball et al., 2013). In addition, MSC-based therapies have been evaluated in inflammatory lung diseases including acute respiratory distress syndrome (Wilson et al., 2015). Where early clinical studies have suggested favorable safety profiles and potential anti-inflammatory effects.

In contrast to MSC therapy, clinical evidence regarding the use of EVs in neonatal or pediatric lung diseases is still lacking. To date, no completed clinical trials have evaluated MSC-derived EVs in infants with BPD or other pediatric pulmonary disorders. However, EV-based therapies have recently been investigated in early-phase clinical studies in adult patients with severe inflammatory lung injury, demonstrating encouraging safety profiles and biological activity (Sengupta et al., 2020). These observations have increased interest in EV-based approaches as a cell-free therapeutic strategy that may retain many of the beneficial effects of MSC therapy while potentially improving safety and standardization.

The main clinical studies investigating MSC- and EV-based therapies in neonatal and pediatric lung diseases are summarized in Table 2.

**TABLE 2:** Clinical studies investigating mesenchymal stromal cell (MSC) and extracellular vesicle (EV) therapies in lung diseases relevant to surfactant dysfunction.

Reference	Population/Disease	Study design	Cell source and route	Main findings
(Chang et al., 2014)	9 extremely preterm infants at high risk of BPD	Phase I dose-escalation clinical trial	Human umbilical cord blood-derived MSCs, intratracheal	Treatment well tolerated; no serious adverse events. Reduction of inflammatory cytokines in tracheal aspirates suggesting anti-inflammatory effects.
(Ahn et al., 2017)	Same cohort as Chang et al.	Long-term follow-up study	Human umbilical cord blood-derived MSCs	MSC administration remained safe up to 2 years of age; no tumor formation or treatment-related complications reported.
(Ahn et al., 2021)	Preterm infants at risk of severe BPD	Randomized phase II clinical trial	Umbilical cord-derived MSCs, intratracheal	Suggestive reduction in severe BPD incidence, particularly in extremely immature infants; overall safety confirmed.
(Pelizzo et al., 2023)	Pediatric patient with surfactant protein C mutation	Case report	Bone marrow-derived MSCs, repeated intravenous infusions	Well tolerated; progressive improvement in respiratory function and reduced ventilatory support.

BPD: bronchopulmonary dysplasia; MSCs: mesenchymal stromal cells; EVs: extracellular vesicles.

Early clinical studies suggest that MSC therapy is safe and may provide beneficial effects, particularly in reducing inflammation and potentially lowering the incidence of severe BPD in the most immature infants. However, these findings should be interpreted with caution due to the limited sample size of available trials, heterogeneity in study populations and treatment protocols, and variability in timing, dosing, and routes of administration. These limitations currently preclude definitive conclusions on efficacy and highlight the need for larger, well-designed multicenter randomized trials. In addition, important translational gaps remain, including variability across preclinical models, lack of standardized manufacturing and characterization protocols, and the still limited and early-stage clinical evidence.

## 6 Limitations

Despite the growing interest in regenerative strategies for neonatal and pediatric lung diseases, several limitations still affect the current body of evidence regarding the use of MSCs and their EVs.

First, most available data derive from preclinical studies**,** primarily conducted in rodent models of hyperoxia-induced BPD. Although these models reproduce several aspects of neonatal lung injury, they do not fully capture the complex pathophysiology of lung disease in extremely preterm infants, which involves multiple interacting factors including mechanical ventilation, inflammation, infection, and developmental immaturity. In addition, differences in experimental design, animal models, cell sources, dosing regimens, and routes of administration make direct comparison between studies challenging.

Second, the translation of preclinical findings into clinical practice remains limited**.** Only a small number of early-phase clinical trials have evaluated MSC therapy in preterm infants at risk of bronchopulmonary dysplasia, and the available studies generally involve small patient cohorts. Although these trials have demonstrated encouraging safety profiles, robust evidence of long-term clinical efficacy is still lacking.

Another important limitation concerns the heterogeneity of MSC sources and manufacturing protocols**.** MSCs can be isolated from different tissues, including bone marrow, umbilical cord blood, placenta, and adipose tissue, and variations in isolation procedures, culture conditions, and expansion methods may significantly influence their biological properties and therapeutic potential. The lack of standardized protocols complicates the comparison of results across studies and represents a major challenge for clinical translation.

Similarly, the development of EV-based therapies faces important methodological challenges. EV isolation, characterization, quantification, and storage methods vary widely across studies, and consensus guidelines for EV production and quality control are still evolving. Furthermore, key aspects such as optimal dosing, biodistribution, pharmacokinetics, and long-term safety remain incompletely understood.

Finally, although regenerative approaches are promising, neonatal and pediatric lung diseases are biologically heterogeneous. Surfactant deficiency may arise from prematurity, inflammatory injury, or genetic disorders affecting surfactant metabolism. Consequently, future studies will need to better define the patient populations most likely to benefit from MSC- or EV-based therapies, as well as the optimal timing and route of administration.

## 7 Future perspectives

Future research should focus on addressing the current limitations that hinder the clinical translation of MSC- and EV-based therapies for surfactant-related lung diseases. A deeper understanding of the mechanisms through which MSCs and their EV interact with the developing lung is required, particularly regarding their effects on ATII and the regulation of surfactant homeostasis. In parallel, significant efforts are needed to standardize MSC sources, culture conditions, expansion protocols and EV isolation methods in order to ensure reproducibility, quality control and scalability for clinical applications.

Advances in EV research may further expand the potential of cell-free regenerative therapies. Improved technologies for EV characterization, purification and cargo analysis will help clarify their mechanisms of action and support the development of more consistent therapeutic products. In addition, bioengineering approaches may enable the design of engineered EVs capable of delivering specific molecular cargo, including regulatory RNAs or proteins, directly to injured lung tissue.

From a clinical perspective, well-designed multicenter randomized trials will be essential to determine the efficacy and long-term safety of MSC-based therapies in neonatal and pediatric lung diseases. Future studies should also aim to identify the patient populations most likely to benefit from regenerative therapies and to define the optimal timing, dosing and route of administration during critical stages of lung development.

Finally, the integration of regenerative approaches with current therapeutic strategies, including surfactant replacement therapy and advanced respiratory support, may represent an important step toward personalized treatment strategies aimed at improving lung repair and long-term respiratory outcomes in infants and children with surfactant-related lung disorders.

## 8 Conclusion

Surfactant deficiency plays a central role in several neonatal and pediatric lung diseases, including respiratory distress syndrome, bronchopulmonary dysplasia and genetic disorders affecting surfactant metabolism. Although advances in neonatal care and surfactant replacement therapy have significantly improved survival, current treatments mainly provide supportive management and do not fully address the mechanisms underlying lung injury and impaired lung development.

In this context, regenerative approaches based on mesenchymal stromal cells and their EVs have emerged as promising strategies to promote lung repair. Preclinical studies consistently demonstrate that MSCs and MSC-derived EVs can modulate inflammation, protect the alveolar epithelium and support alveolar and vascular development in models of immature lung injury. Early clinical studies evaluating MSC therapy in preterm infants have reported encouraging safety profiles and preliminary indications of efficacy, particularly in the prevention of severe bronchopulmonary dysplasia. In contrast, clinical data on EV-based therapies in neonatal and pediatric lung diseases are still lacking**,** and their clinical application remains at an early stage, although accumulating experimental evidence supports their potential as a cell-free regenerative strategy.

Taken together, current evidence highlights the potential of MSC- and EV-based approaches as innovative regenerative therapies for surfactant-related lung diseases. Continued translational research will be essential to better define their therapeutic role and to facilitate their future integration into clinical practice.
